# The Epidemiology of Pre-Hospital EMS Treatment of Geriatric Patients in the City of Vienna—An Overview

**DOI:** 10.3390/jcm12020643

**Published:** 2023-01-13

**Authors:** Mario Krammel, Valentin Drahohs, Thomas Hamp, Sabine Lemoyne, Daniel Grassmann, Wolfgang Schreiber, Patrick Sulzgruber, Sebastian Schnaubelt

**Affiliations:** 1 Austrian Cardiac Arrest Awareness Association (PULS), 1090 Vienna, Austria; 2 Emergency Medical Service, 1030 Vienna, Austria; 3 Department of Anaesthesiology and Critical Care Medicine, Hospital St. Poelten, 3100 St. Poelten, Austria; 4 Department of Anaesthesiology, General Intensive Care Medicine, and Pain Therapy, Medical University of Vienna, 1090 Vienna, Austria; 5 Department of Emergency Medicine, Antwerp University Hospital, 2650 Edegem, Belgium; 6 Department of Emergency Medicine, Medical University of Vienna, 1090 Vienna, Austria; 7 Division of Cardiology, Department of Internal Medicine II, Medical University of Vienna, 1090 Vienna, Austria

**Keywords:** geriatric patients, nursing home, pre-hospital care, emergency medicine, emergency medical service

## Abstract

**Background:** The city of Vienna, Austria, has a gradually aging population. Elderly people, over 65 years old and living at home or in nursing homes, frequently use Emergency Medical Services (EMS). However, there is no previous data comparing the EMS utilization of elderly- and non-elderly patients in Vienna. **Methods:** We retrospectively analyzed all EMS incidents in Vienna from 2012 to 2019. Transport- and emergency physician treatment rates, annual fluctuations, and the number of non-transports were compared between elderly (≥65 years) and non-elderly (18–64 years) patients. **Results:** Elderly people accounted for 42.6% of the total EMS responses in adult patients, representing an annual response rate of 223 per 1000 inhabitants ≥ 65 years. Compared to 76 per 1000 inhabitants in patients 18–64 years old, this results in an incidence rate ratio (IRR) of 2.93 [2.92–2.94]. Elderly people were more likely (OR 1.68 [1.65–1.70]) to need emergency physicians, compared to 18–64 year-olds. Nursing home residents were twice (OR 2.11 [2.06–2.17]) as likely to need emergency physicians than the rest of the study group. Non-transports were more likely to occur in patients over 65 years than in non-elderlies (14% vs. 12%, *p* < 0.001). **Conclusions:** The elderly population ≥ 65 years in Vienna shows higher EMS response rates than younger adults. They need emergency physicians more often, especially when residing in nursing homes. The economical and organizational strain this puts on the emergency response system should trigger further research and the development of solutions, such as specific response units dedicated to elderly people.

## 1. Introduction

The highly frequent utilization of emergency medical services (EMS) is known internationally in high-resource settings [1]. Elderly people ≥65 years of age use EMS at a disproportionately high rate [2,3]. The city of Vienna, Austria, faces the well-known issue of population aging. From the roughly 2 million residents, 16.5% were aged ≥65 years or older in 2019. This percentage is predicted to increase up to 22% by 2048. Moreover, the percentage of those aged ≥ 80 years is predicted to double until then [4,5].

Differences in the (patho-)physiological and socioeconomic attributes of the elderly, when compared to younger counterparts, are well-known [6,7,8]. Emergency medicine, in particular, therefore, often poses challenges for medical personnel attending the elderly; indeed, complex situations, including multimorbidity, an increased risk of fall, nonadherence, and communication problems, tend to bind resources [8,9,10]. Together with the growth in numbers of this patient group, emergency departments face an increasing challenge to successfully manage the respective cases. In addition, revisits are frequent, and the EMS is often used by out-of-hospital care facilities to “shift” the medical problems of elderly individuals, for instance, nursing home inhabitants, to the hospital setting [11,12,13,14]. This complex problem was identified long ago [15], but research data and novel interventions are still scarce. Moreover, studies focusing on EMS utilization are rare, especially those centred around elderly patients.

## 2. Materials and Methods

### 2.1. Study Population and Data Acquisition

For this retrospective observational study, we screened all emergency responses by the EMS Vienna, Austria, between 01/2012 and 12/2019. Routine electronic patient records from all ambulances (Basic Life Support, BLS, and Advanced Life Support, ALS, units) with primary emergency tasks were analysed. The basic demographics, reason for the emergency call, location category, emergency physician attendance, transportation destination, and the National Advisory Committee for Aeronautics (NACA; developed to grade the severity of medical or traumatic emergencies; standardly used by Austrian EMS organisations) score [16,17] were extracted. The respective patients, no matter whether they were transported to a hospital or not, or whether they were living at home or in a nursing home, were categorized into elderly (≥65 years; 65 is the retirement age in Austria) and non-elderly (18–64 years). It should be noted that ambulances from private organisations, with the primary task of emergency response, were also part of this analysis; this is because they use the same electronic patient records that are provided by the EMS of Vienna. However, those cases from private organisations, primarily for non-emergency patient transport, were not included. Further exclusion criteria were patients under the age of 18 years, EMS admissions that were different to “classical” emergency responses with one patient (e.g., mass casualty events), admissions of special units (e.g., field supervisors), inter-hospital transfers, and admissions without patient contact.

Population data from the Austrian national statistics office (Statistik Austria) were used to calculate transportation rates per 1000 inhabitants.

Ethical approval for this study was provided by the Ethical Committee of the Medical University of Vienna, Austria (No. 1724/2019). Informed consent was waived. The study protocol complies with the Declaration of Helsinki and data reporting was performed according to the STROBE and MOOSE guidelines.

### 2.2. The Viennese EMS

Austria has a two-tiered emergency medical service (EMS) with basic, advanced, and additionally skilled emergency medical technicians (EMT), as well as emergency physicians for critically ill patients. BLS-providing units are staffed with at least two basic EMTs, whereas ALS-providing units in Vienna include at least one advanced EMT. In Austria, pre-hospital emergency medicine is not a physician speciality, but an advanced training module for medical doctors. Physician-staffed units in Vienna are usually hospital-based and dispatched either in parallel to an ambulance unit or additionally, if the EMTs on scene call for support. The Emergency Medical Service Vienna (MA 70) is part of the city of Vienna and is the largest EMS provider in Vienna. However, six private organisations, for instance, the Viennese Red Cross, also provide ALS- and BLS-units to support the MA70. Furthermore, these private organisations also operate BLS-units used for non-emergency patient transport.

The EMS in Vienna also operates the emergency dispatch centre with the medical emergency number 144. The dispatch centre uses the Advanced Medical Priority Dispatch System (AMPDS) for standardized call-taking and was certified as an accredited Centre of Excellence by the International Academies of Emergency Dispatch in 2021.

The threshold for people calling the emergency number and requesting an ambulance is low because there is no direct user fee.

### 2.3. Statistical Analysis

We processed all data in Microsoft Excel 2013 (Microsoft Corporation, Redmond, WA, USA), and performed a statistical analysis using SPSS 22.0 (IBM, Armonk, New York, USA). Raw counts, percentages, means and the standard deviation (SD) were used for descriptive characteristics in both age groups and the total study population. Transport rates were calculated by the total number of attendances, divided by the totalized population over the 8-year period; this was converted into transports per 1000 inhabitants, respectively, for each age group. We performed a T-test for age, a Mann–Whitney–Wilcoxon test to the compare annual growth in relation to the previous year, and χ^2^-tests for categorical variables to evaluate the difference between the elderly and non-elderly. The statistical significance was defined by two-tailed *p*-values of <0.05.

## 3. Results

### 3.1. EMS Responses

There were 1,277,172 EMS responses for patients ≥ 18 years over the 8-year period, representing an average of 159,647 cases annually. Whilst the population of Vienna grew by 10.0% [9.9–10.1] in 2019, when compared to 2012, EMS responses only increased by 0.8% [0.7–0.8] (*p* < 0.001). Thus, the overall response rate decreased from 108 in 2012 to 99 responses per 1000 inhabitants in 2019. This represents an average annual decline of 1.2% [1.2–1.2]. The transportation rate for elderly patients decreased from 224 to 217 per 1000 inhabitants from 2012 to 2019, and, therefore, the decline in the transportation rate is less than in adults under 65 years old (78 to 69 per 1000 population from 2012 to 2019); however, this result was not significant (*p* = 0.401).

As for the reason to call EMS, medical conditions were the most common reason for EMS missions (66% overall). Trauma was more common in patients 18–64 years (*p* < 0.001). Most patients were rated with a NACA (see Methods) score II, though a NACA score ≥ III was more frequent in elderly patients (*p* < 0.001) (Table 1).

### 3.2. Age and Gender

**Figure 1:**
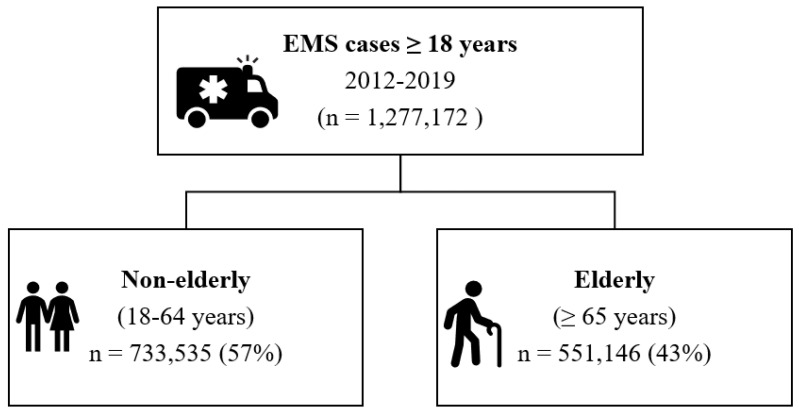
Study flow chart. EMS = Emergency medical system.

**Figure 2:**
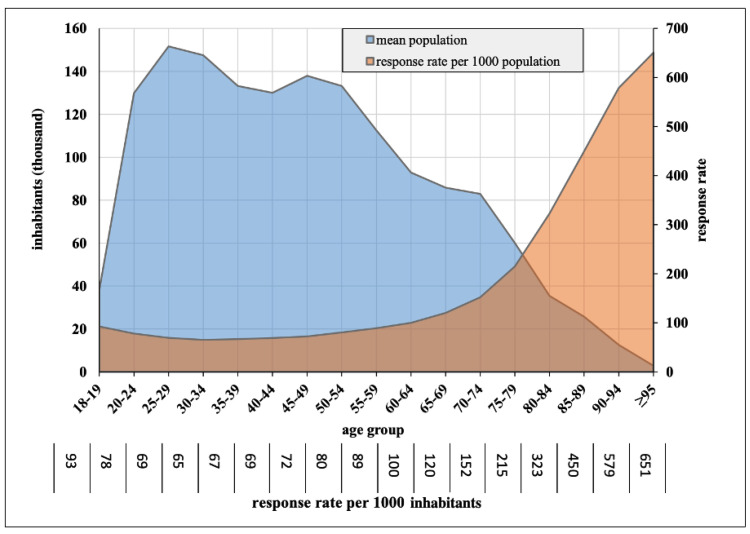
EMS response rates in Vienna by age group (2012-2019).

Among the 1,277,172 adult emergency responses, the EMS was called for elderly patients in 43% of cases (Figure 1). Patients ≥ 65 years were three times (incidence rate ratio (IRR) 2.93 [2.92–2.94]) as likely, and patients over 80 years were five times (IRR 5.53 [5.50–5.55]) as likely to initiate an EMS response, compared to the younger reference group. Accordingly, higher age was associated with an exponentially rising response rate, starting at an age of approximately 60 years (Figure 2). Overall, women represented 108 responses per 1000 inhabitants, compared to 103 responses per 1000 inhabitants for men (IRR 1.05 [1.04–1.05]). However, when age was also considered, the IRR varied from 0.93 [0.93–0.97] to 1.02 [1.02–1.03] for younger adults and the elderly, respectively.

### 3.3. Emergency Physicians

**Figure 3:**
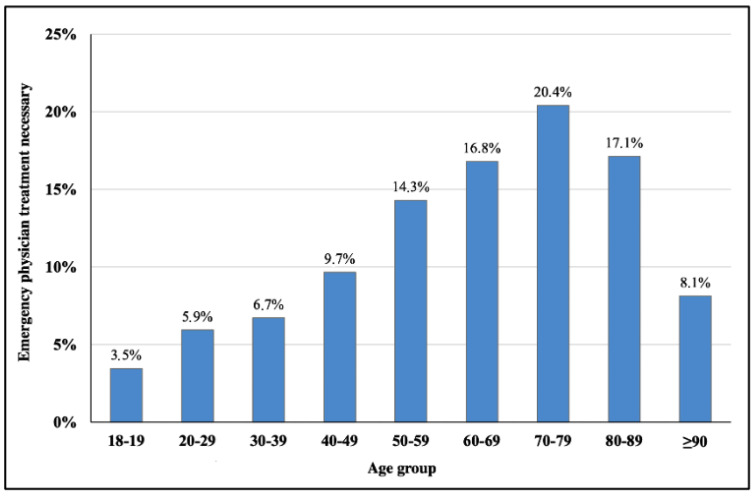
Therapy by prehospital emergency physicians in Vienna by age group (2012–2019).

Emergency physicians performed therapy (any kind) in 68,227 (5.3%) of the total responses, representing an overall prehospital emergency physician treatment rate of 5.6 per 1000 inhabitants. Elderly people ≥65 years were around 1.5 times (OR 1.68 [1.65–1.70]) more likely to need an emergency physician when compared to their younger counterparts. The emergency location category had a significant influence on the attendance of emergency physicians (Figure 3), showing about twice (OR 2.11 [2.06–2.17]) as many prehospital treatments in nursing homes than any other location.

### 3.4. Transport and Non-Transport

Most patients (87% overall) were transported into a hospital. However, non-transports were more frequent in patients over 65 years than in younger ones (14.0% [13.9–14.1] vs. 12.0% [95%-CI 11.9–12.1], *p* < 0.001). The main reason for non-transports was the patient refusing transport in 66.3% [66.1–66.6] of cases, either signing a letter of indemnity or on the advice of an EMT or physician.

## 4. Discussion

In summary, elderly people accounted for around 43% of the total EMS responses of adult patients in Vienna. Elderly people were more likely to need emergency physicians compared to 18–64 year-olds, and nursing home residents were twice as likely to need emergency physicians than the rest of the study group. Non-transports were more likely to occur in patients over 65 years than in non-elderlies.

### 4.1. Response Rates in International Comparison

To our knowledge, this is the first study to research the epidemiology of the metropolitan EMS utilization of elderly patients in Austria. Response rates were in the upper range when compared to other developed countries [2,18,19,20,21,22]. However, the response rates of the younger counterparts should also be considered, due to different EMS systems, depending on different countries, urban or rural locations and high- or low-resource settings. The IRR for elderly, as opposed to younger adults, was lower than in previous studies (Canada: 4.1, Taiwan: 7.1, USA: 4.3) [2,18,21]. Furthermore, the overall response rate in Vienna was twice as high when compared to, for example, Melbourne in 2007 (107 versus 58 per 1000 inhabitants) [3]. Tokyo has a similar IRR for elderly patients, but also a lower overall response rate of 55 responses per 1000 inhabitants [20]. When compared to the nearby Bavaria or northern Italy, we found comparably high overall response rates (70 and 102 per 1000 inhabitants) [6,23]. This shows an internationally high usage of the EMS by elderly people, but also suggests a generally high EMS utilization, at least in Europe.

### 4.2. Low Urgency Missions

Approximately 10% of patients were rated NACA (see Methods) I, and the EMS responses can, therefore, be considered of minor urgency. More than half of the responses were rated NACA II, which, therefore, could potentially have been handled by general physicians or primary health care centres instead of the EMS. This shows a partly inconsiderate use of emergency resources, and a potentially impaired access to primary care. From our data, one might deduct that younger patients more often induce an EMS response with a lower urgency (NACA I or II); however, further research is needed to examine the reasons for low-priority EMS calls, especially those rated NACA I and II.

### 4.3. A growing Strain on the System

Overall, non-transport missions were less common than in other studies (Taiwan: 22.6%, Canada: 18.2%) [18,21]. We found non-transport in 12.8% of cases in this study; however, when other reasons for non-transport, except patients refusing transport, were excluded, this occurred in only 8.4% of the responses. Younger adults had non-transports approximately twice as often compared with the elderly in previous studies, which we could not confirm in Vienna; our data showed non-transports in elderly patients appearing in 14.0%, which is comparable to 12.3% in Australia and 12.0% in Taiwan and Canada [18,21,22]. Thus, many patients are transported to the hospital, meaning that there may potentially be a risk of inappropriate care. Prehospital emergency physician treatment rates were 5.6 per 1000 inhabitants overall. Although the rates vary depending on the emergency location, we found lower rates in Vienna than in Germany (ranging from 16.9 to 21.5 per 1000 population) [6,23,24]. These results leave us without a clear statement; it could be that in Vienna elderly patients are often transported to a hospital, even when the medical condition is not very clear; this could be because the situation, potentially including many co-morbidities and impaired communication, might often be more complex in elderly patients than in cases with younger patients. In addition, a “social indication” for transportation might be existent in a number of situations, e.g., when the EMS does not feel comfortable leaving the elderly patient in an inferior state of care (e.g., alone at home), even if no medical emergency is present. Together with the fact that emergency physician treatments were significantly more frequent in the elderly, especially in nursing homes, we highlight concerns regarding the immense economic and organizational strain the large (and growing!) group of elderly patients puts on the emergency response system [14]. One can thus ask whether EMS interventions are really always the most suitable form of care for this vulnerable patient group. Further endeavours should be undertaken to address this matter, for instance, through improved outpatient care from general practitioners, the development of specific units responding to nursing home emergency calls, increased capacities for specialized geriatric facilities, or improved hospital discharge plans [25,26,27,28,29,30].

### 4.4. Limitations

This study had considerable limitations; these included incomplete data regarding unknown age (5.5% of cases) and, most notably, responses by various private organisations that could not be included in this study. This occurs, for example, when employees of nursing homes do not call the emergency number when they need an ambulance for a patient, but instead the private service numbers of these organisations. Another reason could be elderly people using home-emergency buttons, operated by said organisations. It can be assumed that the major number of responses by private organisations is, indeed, for the elderly population; therefore, response rates for elderly patients might actually be even higher than reported here. Another limitation is that we do not have detailed information about the kind of therapy provided by the respective emergency physicians or about hospital admission/discharge and/or respective diagnoses. This study was performed retrospectively and in a single, high-resource region. Additionally, we only performed quantitative analyses. Thus, the generalization of our findings to other settings and systems is limited.

Of note, this topic, focusing on age-specific differences, has been assessed before from an in-hospital point of view (e.g., emergency department visits) [31]. Our study focused on the pre-hospital situation and can, therefore, not be applied to in-hospital conditions.

## 5. Conclusions

The elderly population ≥65 years old in Vienna shows higher EMS response rates than younger adults. They need emergency physicians more often, especially when residing in nursing homes. The economical and organizational strain this puts on the emergency response system should trigger further research into this topic and the development of solutions, such as specific response units dedicated to elderly people.

## Figures and Tables

**Table 1:** Characteristics of elderly versus non-elderly patients in demand of EMS care between 2012 and 2019 in Vienna.

		Total *n* = 1,277,172	Non-Elderly (18–64 Years) *n* = 733,535	Elderly (≥65 Years) *n* = 551,146	*p*-Value
Annual responses, *n*		159,647	91,692	67,955	**<0.001**
Response rate, *n* per 1000 population		105.6	76.0	222.6	**<0.001**
Age, years (±SD)		57.2 ± 22.2	41.0 ± 13.6	79.2 ± 8.4	**<0.001**
Female, *n* (%)		681,003 (53)	357,520 (49)	323,483 (60)	**<0.001**
Emergency physician treatment, *n* (%)		68,227 (5)	30,889 (4)	37,338 (7)	**<0.001**
Emergency physician treatment rate, *n* per 1000 population		5.6	3.2	15.3	**<0.001**
Transport to hospital, *n* (%) *		1,113,119 (8.7)	645,523 (88)	467,596 (85)	**<0.001**
Non-Transport, *n* (%) *		163,990 (13)	87,979 (12)	76,011 (14)	**<0.001**
Reason of call, *n* (%)	medical condition	840,673 (66)	466,028 (64)	374,645 (69)	**<0.001**
	trauma	271,395 (21)	165,375 (23)	106,020 (20)	
	other	164,562 (13)	101,769 (14)	62,793 (12)	
NACA score, *n* (%)	I	127,094 (10)	76,067 (10)	51,027 (9)	**<0.001**
	II	810,359 (64)	506,566 (69)	303,793 (56)	
	III	285,347 (22)	128,002 (18)	157,345 (29)	
	IV	29,357 (2)	13,609 (2)	15,748 (3)	
	V	7405 (0.6)	3310 (0.5)	4095 (0.8)	
	VI	3758 (0.3)	1720 (0.2)	2038 (0.4)	
	VII	13,323 (1.0)	4108 (0.6)	9215 (1.7)	

NACA **=** National Advisory Committee for Aeronautics. * The difference to 100 percent is due to the category “others/unknown/multiply mentioned”.

## Data Availability

The data presented in this study are available on request from the corresponding author. The data are not publicly available due to national legislation.
